# The impact of online office on social anxiety among primary and secondary school teachers—Considering online social support and work intensity

**DOI:** 10.3389/fpsyg.2023.1154460

**Published:** 2023-03-30

**Authors:** Yating Xie

**Affiliations:** ^1^College of Educational Science, Hunan Normal University, Changsha, Hunan, China; ^2^College of Fine Arts and Design, Changsha Normal University, Changsha, Hunan, China

**Keywords:** online social support, online office, social anxiety, structural, equations models

## Abstract

The COVID-19 has had a major impact on the global education system. In order to ensure the normal implementation of education courses, governments and education departments around the world have taken corresponding emergency measures. Based on data from 384 validated questionnaires, this study explored the effects of teleworking practices, work intensity, and online social support on social anxiety among primary and secondary school teachers. The results found that teleworking was more likely to cause social anxiety among teachers, while work intensity could promote social anxiety and online social support could reduce the probability of social anxiety. Work intensity can weaken the influence of partner support on social anxiety. Moreover, the model path coefficients differed across work styles. Based on the results, this study proposes some policy recommendations in order to provide theoretical guidance for improving social anxiety among primary and secondary school teachers and promoting the quality of educational work.

## 1. Introduction

The respiratory disease caused by coronavirus was first identified in Wuhan, China, in 2019, initiating the largest pandemic of the 21st century. on 30 January 2020, the World Health organization officially declared COVID-19 a public health epidemic. In the months that followed, cases were reported in countries around the world, threatening global health. The global epidemic of COVID-2019 posed unprecedented challenges to security, health, education, economy, and job stability (Andrade-Vargas et al., 2021). In order to prevent the rapid spread of the virus, various countries have to take corresponding emergency measures. The World Health Organization recommended the wearing of masks in public and maintaining appropriate social distancing to prevent the spread of the virus (Yan-Rong et al., 2020). In this case, classrooms and schools are considered high-risk Spaces for the transmission of the virus due to the large and concentrated population. Therefore, more than 90% of countries choose to temporarily close their teaching areas and adopt the way of distance learning to carry out education and teaching (Basilaia and Kvavadze, 2020). These changes are based on the implementation of emergency education and virtual distance education. In order to ensure that students in different situations can attend classes normally, education and teaching work needs to be more flexible. In addition, the World Bank has begun working with national education authorities to support them in adopting a virtual education approach in response to the global crisis. Other international organizations such as the United Nations Children’s Fund (UNICEF) have since joined this initiative. In the face of the virus, every country is looking for alternatives to offline education, all of which require, to a greater or lesser extent, the use of virtual education models. For example, during the New Coronary Pneumonia epidemic, China launched the One-Stop Learning initiative. The goal of the initiative was to develop a systematic program to address the COVID-19 pandemic and the main issues related to the public health crisis. The Chinese government has taken measures to: a. promote online education, b. train teachers, c. develop academic research centers, and d. organize logistical operations. The ultimate goal of the program is to achieve uninterrupted learning.

There is no doubt that under this reality, there will be a series of changes in the education system, and teachers will be the most affected group (Faisal Mahmood et al., 2021)^.^Due to the closed management of major schools, offline education was announced to be suspended, while the education plan still needs to be carried out, the education department has to change the teaching model and carry out remote teaching (Unger and Meiran, 2020). In effect, from 1 day to the next, teachers, students, and their families were constantly trying to adapt to this change. In the case of teachers, they have had to fundamentally change the way they work, move to online office without realizing the increased demands of their jobs, or stop to think about whether they have the resources necessary to cope with the dramatic changes that come with forced home teaching. Remote working can improve job satisfaction, increase autonomy, and reduce work–family conflict among remote workers to some extent (Bakker, 2007) and the impact of teleworking on energy savings in an organizational setting (Hook et al., 2020). Similarly, negative effects of teleworking have been reported, such as social isolation or coworker dissatisfaction (Golden et al., 2008).

Personally, not everyone has the same resources or has received the same training to meet challenges. Some teachers had experience in distance training before, but others did not. Some of them have handled the new technology well (maybe young teachers), while others have difficulty (definitely older teachers). A successful tele work plan should specify the responsibilities of employees and employers, tele employee agreements, working hours, employee expenses (reimbursable and non reimbursable), maintenance and recovery procedures for tracking equipment, furniture and other assets, training requirements and schedules. Therefore, this will be a process of analysis and evaluation before implementation. However, this description bears little resemblance to what happened in the first weeks of the pandemic. Suddenly, teachers are forced to work remotely, although in reality, it is more like working at home, closing your eyes and crossing your fingers, a kind of “doing what you can do” without analyzing or evaluating the consequences. Scientific literature has seen the advantages and disadvantages of teleworking.

Existing studies have explored several aspects of teleworking on teachers’ physical and mental health (Barros, 2017), family harmony (Paschoal et al., 2022), work engagement (Lewig and Metzer, 2007), job satisfaction (Gu et al., 2020), and job well-being (Gajendran and Harrison, 2007), the results of the study showed different effects depending on the region, environment, and group. However, the effect of teleworking on teachers’ social anxiety has not received the attention it deserves, and there are almost no relevant studies. People with social anxiety show a pronounced and persistent fear of various social scenarios or appearing in social events that make people embarrassed, and once they are exposed to such situations, they feel overwhelmed and experience various symptoms of discomfort (Piccirillo et al., 2016). Teachers have made great contributions to the inheritance of human culture and the development of scientific knowledge. With the help of teachers, students can better shape their personality and enhance their ability. The quality of teachers’ work is closely related to the physical and mental development of students and the progress of the country. Social anxiety not only has a negative impact on teachers’ physical and mental health, but also seriously affects teachers’ work quality (Samantha et al., 2020). Although there are no studies examining the relationship between teleworking and teacher social anxiety, teleworking can reduce opportunities for face-to-face interaction between teachers and teachers and between teachers and their peers. Some studies have shown that long-term home isolation can negatively affect people’s mental health, leading to panic, anxiety, depression, frustration, anger, boredom, and paranoia (Samantha et al., 2020; Souvik Dubey, 2020). However, it is unknown whether teleworking leads to social anxiety among teachers. Therefore, it is important to explore the effects of teleworking on teachers’ social anxiety.

The purpose of this study is to explore the effects of remote work style on social anxiety of elementary and secondary school teachers, taking into account online social support and work intensity. In order to provide more insight into the mechanisms of the effects of remote work styles, this study will compare the differences in teachers’ social anxiety between remote and non-remote work styles and explore the differences in the effects of online social support and work intensity on social anxiety in the two work styles. The findings of this study aim to enhance the well-being of primary and secondary school teacher populations and promote sustainable development in education.

The theoretical significance of this study is as follows: based on the special environment affected by the epidemic, this paper aims at the mechanism of remote work of primary and secondary school educators, and gives a reasonable explanation for the relevant mechanism, thus further expanding the research on the theory of social anxiety.

The practical significance of this study is as follows: Based on the background of the epidemic, it is difficult for primary and secondary school education to use offline conditions to carry out education work, so as to use the network environment to carry out education work, to study the influence of social support and other factors on the social anxiety of primary and secondary school educators under the new working environment, and to explore the deterioration of the adaptation of primary and secondary school educators to the new working environment. Effective measures to effectively relieve social anxiety, so as to improve the adaptability of primary and secondary school educators to the new teaching environment, effectively relieve social anxiety, improve work efficiency, and provide effective coping strategies, has a strong practical guiding significance.

## 2. Literature review

As a result of the ongoing impact of the pandemic and the increasing use of technological advances and information technology, telework is gaining popularity and attracting widespread interest from academic scholars, administrators and organizations, but there is no exact definition of telework. Remote workers are widely accepted in academic circles and are defined as people in dependent employment relationships who use information technology and communications as a means to carry out work from the employer’s location in any form prescribed by law. In a world where remote work is not just a shrunken call center, neither working from home nor temp services, but a viable and legal alternative to employment; It requires open mindsets and paradigm shifts in organizations, managers, and remote workers, as well as flexibility in thinking, schedules, work environments, and work outcomes. That is, it requires plasticity and adaptability to emerging space–time models. Remote working is a form of work that has both benefits and risks. One of these advantages has to do with the macro environmental systems that affect humans. It can be noted that the corresponding advantages point to a large extent to various aspects of a person’s mental health, namely his emotional dimension, social, family and cognitive. In fact, talking about isolation, loneliness, stress, mood, reduced social and work relationships, insecurity, etc., are all considered predictive variables of threatening health. Although the 21st century can be considered as the era of online education (Cutri and Mena, 2020), due to the rapid changes in technology and adoption of educational technologies (Sokal et al., 2020; Tempski et al., 2020), many developing countries are still using offline education as the main method, and most of the face-to-face education models that are used to online teachers are not adequately prepared for online education (Mahmoud Al-Balas et al., 2020).

This teleworking environment for teachers led to various consequences of health conditions due to overload during the pandemic, including depression, anxiety, stress, and burnout syndrome (Leire Aperribai et al., 2020). The impact of online office on Chilean teachers during the 2019 coronavirus disease pandemic was reported to exacerbate the production of anxiety and stress, resulting in high workload, exhaustion, and burnout (Pablo and Lizana, 2021).In addition, prior to the COVID-19 pandemic, one of the occupations with the greatest global health deterioration was teaching (Li, 2020), with a significant increase in mental health deterioration and physical discomfort in professional practice, resulting in psychosocial deterioration and occupational stress (Bauer et al., 2007; Lizana et al., 2020)^a^nd burnout (Arvidsson et al., 2016) resulting in quality-of-life impairment. Although a large number of studies have analyzed the impact of teleworking on teachers’ physical and mental health after the COVID-19 pandemic, they have neglected teachers’ social anxiety status. Comparing teleworking styles with non-teleworking styles, as opposed to setting teleworking styles as an independent variable, better captures the mechanism of the effect of teleworking styles on teachers’ social anxiety, which is one of the main innovations of this study.

In addition, social anxiety, as a common emotional problem in individuals’ interpersonal interactions, is affected by physiological, psychological, and social factors, and also has an effect on individuals’ emotions, cognition, and behavior (Lpine et al., 2000). With the development of the times, online social support has become a new way to support the development of the real society, and between the anonymity and the stronger interaction of the network itself, more and more people are involved in online social support or find confidence in the network (Cherba et al., 2019). In the academic field, research on social support can be traced back to the late 19th century French sociologist Dürkheim, who found in his study of suicide that the closeness of social ties was associated with suicide rates and that the loss of social ties/support contributed to one of the major causes of suicide. In the 1970s, social support was formally introduced as a scientific term. Social support refers to the love, respect and concern that individuals feel when interacting and communicating with others. This supportive interaction helps individuals offset the negative impact of life pressure on health. Social support is a concept that encompasses multiple structures and has been defined differently by researchers from different perspectives. Broadly speaking, however, social support refers primarily to the material and emotional help that people receive from social groups, organizations and various social relationships. In terms of the sources of social support, it includes both formal and informal social support. Formal social support generally refers to state support embodied by national policies and regulations, support from units that specifically implement national policies and regulations, as well as support from other community organizations and civil society organizations; informal social support refers to support from various social relationships, such as family, relatives, classmates, friends and informal social support refers to support from various social relationships, such as family, relatives, classmates, friends, colleagues, etc.

As a complement to the type of social support, online social support is a meaningful development of real social support and adds a new channel for individual interpersonal interaction. Online social support consists of four dimensions: companion support, instrumental support, information support, and emotional support (Nick et al., 2018). The biggest differences between real social support and online social support are the characteristics of online anonymity, visual absence, lack of temporal and spatial barriers and elastic synchronization. Real social support in which the supporter and the recipient generally need to exchange personal and real information, instrumental support is required for the supporter to alleviate the bondage of the real situation with full knowledge of the recipient. Online social support does not require face-to-face communication and getting along, and for individuals with weak interpersonal social skills, it can avoid the stresses associated with interpersonal interactions while not interfering with interactions with others. Group diversity is a key feature of online social support. Whereas most real social support is only recognized by peers in their own sphere, expressing oneself online allows one to interact with many types of people, exchange ideas and gain the approval and support of others. Also, one of the key reasons why individuals tend to express themselves online is because they can choose not to reveal their real names in online interactions, which makes the content of interactions between individuals more private. Some studies have shown that individuals who are more shy in real life will prefer online interactions as a way to compensate for the deficiencies that come with real interactions.

The study showed that online social support was significantly and negatively associated with social anxiety and significantly and negatively predicted social anxiety (Ruppel and McKinley, 2015). According to social cognitive theory, individuals with low levels of social support are blunt and do not easily experience pleasure in social interaction activities, they experience less social support subjectively, perceive social activities as low value and they do not get attention and rewards from them, and the resulting unpleasant experience leads to social anxiety (Piccirillo et al., 2021). The buffer model of social support suggests that when individuals encounter stressful events or situations, social support acts as a buffer to cushion the consequences of the stressful event or situation, promoting better stress reduction and health maintenance (Wang and Zhang, 2021). When individuals have higher levels of perceived social support, they are also motivated to have more confidence in social situations, and this perceived social support can buffer individuals from possible stress and anxiety in social situations, thereby reducing social anxiety levels (Porter and Chambless, 2017).

In addition, work intensity affects teachers’ physical and mental health, but existing studies have ignored the relationship between work intensity and social anxiety. This complexity exacerbates existing inequalities in access to quality education and has generated high levels of stress, anxiety and general discomfort among teachers as a result of changes in teaching and learning styles and an epidemic that widens the economic, structural and capacity heterogeneity of schools and teachers. The above studies suggest that lack of connections and adequate digital skills in online teaching are associated with stress and poor mental health.

Based on the above studies, the following research hypotheses are proposed:

*H1*: Work intensity has a significant positive effect on teachers' social anxiety.

And that peer support and better coping strategies are protective factors. In this sense, recent studies have shown that the instability, uncertainty and fear caused by the COVID-19 pandemic are worryingly exacerbating poor mental health. Poor mental health is a widespread problem that affects not only patients but also their families, work environments and education systems. As noted above, the implementation of mandatory telework without proper working conditions may affect the mental health or well-being of teachers and, in turn, the quality of education they provide to their students. We identified gaps in the literature on the mental health of teachers working remotely in the context of the pandemic. For these reasons, we believe it is necessary to understand the mental health status of teachers, paying particular attention to experience, as inexperience appears to be a factor influencing higher mobility and gender, as women may have additional workloads in domestic or child care based on stereotyped gender roles, which may be exacerbated during the pandemic (Unger and Meiran, 2020).

Based on the above studies, the following research hypotheses are proposed:

*H2*: Companion support has a significant negative effect on teachers' social anxiety.

Jiezong (2014) pointed out that in the environment of information society, human existence and life style have undergone radical changes. In the field of education, modern information technology is being used more and more widely. However, the application of information technology can change the teaching methods of teachers, so that teachers can use information technology to carry out teaching independently from the specific actual teaching environment, and greatly reduce the opportunity for teachers to contact with others, thus aggravating the anxiety of teachers.

Based on the above studies, the following research hypotheses are proposed:

*H3*: Information support has a significant negative effect on teachers' social anxiety.

Unger and Meiran (2020) put forward that there are many factors affecting the optimization of teaching process, and teachers’ emotional support is one of the most important factors. This factor affects teachers’ social anxiety in the teaching process and thus affects teachers’ teaching state. Piccirillo et al. (2016) studied the relationship between safety behaviors and social anxiety of adults and believed that social anxiety of adults in teaching and other professions had an inverse relationship with their emotional support (Unger and Meiran, 2020).

Based on the above scholars’ research on the influence of emotional information support on teachers’ anxiety, the following hypotheses are proposed:

*H4*: Emotional support has a significant negative effect on teachers' social anxiety;

Liang and Chen (2021) found through investigation and analysis that instrumental support of teachers in teaching can promote the improvement of teachers’ teaching effect, and teachers will become dependent on it, which makes them easy to deviate from normal social activities and thus increase their anxiety.

Based on the above scholars’ research on the influence of emotional information support on teachers’ anxiety, the following hypotheses are proposed:

*H5*: Instrumental support has a significant negative effect on teachers’ social anxiety.

In general, work intensity should be reflected in two ways: the physical or mental exertion that workers incur per unit of time because of their work, and the length of time that workers work. The former is more difficult to measure and analyze, so scholars usually judge whether people are working too hard, mainly based on whether workers work too long hours (Panter-Brick, 2003). One study discusses this trend through the key dimensions of working time (length, duration, pace, and autonomy) and considers the role of current trends in changing workspaces. Changes in working time patterns are driven by several drivers: globalization and business restructuring that challenge current work organizations, new information technologies, demographic and climate change, and current and future pandemics. The dramatic changes in working hours, past and present, suggest that changes in working hours will continue. Contemporary trends in future working time pose risks to personal, family and social life, material well-being and health. Excessive work intensity not only directly and negatively affects teachers’ physical and mental health, but also, working long hours inevitably shortens teachers’ leisure time and social time, leading to social anxiety and thus causing secondary damage to physical and mental health. When the work intensity is high, teachers’ energy and time are largely occupied by work, which will weaken the impact of online social support on social anxiety. Although there is no research evidence to prove this conclusion, the reality seems to be so.

Based on the above analysis, the following hypotheses are proposed in this study.

*H6*: Work intensity has a negative regulatory effect between companion support and social anxiety;

*H7*: Work intensity has a negative regulatory effect between information support and social anxiety;

*H8*: Work intensity has a negative regulatory effect between emotional support and social anxiety;

*H9*: Work intensity has a negative regulatory effect between instrumental support and social anxiety.

In addition, some scholars theoretically analyzed the gap between offline and online teaching.

Yang et al. (2022) analyzed the gap between online and offline education and believed that the advantage of offline education lies in that teachers and students can feel the real classroom atmosphere, have face-to-face contact and solve relevant problems efficiently. Online teaching can be widely used in a variety of practical teaching tools, greatly enriching the teaching technology and means.

Zhang (2022) put forward his own views on the differences between “online” and “offline” education. He believes that there are obvious differences between the offline and online teaching methods suitable for different subjects or courses. Therefore, teachers need to reasonably choose appropriate teaching methods according to different courses. During the epidemic period, some courses that were originally more suitable for offline teaching had to be carried out online due to the impact of the epidemic, which greatly affected the learning effect of students.

## 3. Method

### 3.1. Participants

Primary and secondary school educators in Sichuan, China, were selected for this study，and take it as a whole of the study. Many primary and secondary schools in Sichuan, China, were unable to teach offline due to the epidemic, and staff members were converted to online office from home. After detailed questionnaire design, validation, and testing, data were collected from primary and secondary school teachers through an online format. A total of 429 questionnaires were collected, and 384 questionnaires (222 online office and 162 in the office) remained after eliminating invalid questionnaires, with a validity rate of 89.51%. Among the respondents, 259 (67.4%) were female and 125 (32.6%) were male; age was mainly concentrated in 30–40 years old (216, 56.3%), 48 (12.5%) in 20–30 years old, 47 (12.2%) in 40–50 years old and 73 (19.0%) in 50–60 years old; monthly income was less than 3,000 RMB 8 (2.1%), 121 (31.5%) for 3,000–5,000 RMB, 174 (45.3%) for 5,000–7,000, 49 (12.8%) for 7,000–10,000 RMB, and 32 (8.3%) for over 10,000 RMB.

The reason why we choose online format to collect data from primary and secondary school teachers is that, first of all, the offline activities of primary and secondary school educators are restricted during the epidemic period, and they cannot freely accept the phenomena interviewed by the investigators. Meanwhile, for the investigators, the phenomenon investigation activities are also restricted, and the participants cannot be surveyed offline in a face-to-face way. Therefore, participants were surveyed in an online format; Secondly, due to the large number of survey objects, even if the offline survey can be adopted during the epidemic period, due to the wide distribution of participants, investigators have limited time to investigate. Therefore, the offline survey requires a lot of time, which makes the efficiency of the survey extremely low. Therefore, the online survey is adopted.

The determination of the total number of people surveyed. The purpose of this study is to explore the influencing factors of online office social anxiety of primary and secondary school educators under the epidemic environment, as well as the specific effects of related factors on social anxiety of primary and secondary school educators, and to obtain a universal research conclusion. Therefore, in order to improve the representativeness of the study, When conducting a questionnaire survey, a large number of participants are required to participate. Only in this way can the persuasiveness of this study be improved. At the same time, according to the basic requirements of statistics on sampling survey data collection and statistical analysis, in order to ensure the scientificity and rationality of sampling survey analysis results, large sample data is required when determining the sample size of large-scale population sampling survey, so as to ensure the representativeness of sample data to the research population. And to ensure the rationality and reliability of sampling survey and statistical analysis of data; At the same time, considering that some participants’ filling in the questionnaire may not meet the basic requirements of the survey and questionnaire analysis, it is necessary to eliminate them, so as to appropriately increase the understanding of the survey participants. Therefore, the total number of survey participants is determined to be 429 in this paper.

## 4. Measures

### 4.1. Social anxiety

The main measurement tool for social anxiety is a self-rated scale, which is used to understand the level of social anxiety of individuals by assessing their cognitive, somatic, and behavioral situations in social scenarios. The Social Anxiety Scale (IAS) developed by Leary (1983) was selected for this study to measure the level of anxiety in interpersonal interactions. The scale contains 15 items and is scored on a five-point scale (1 being “not at all” and 5 being “extremely”), with a minimum score of 15 and a maximum score of 75. The higher the score, the higher the social anxiety level of the subject; the lower the score, the lower the social anxiety level of the subject. SA3 (I usually feel relaxed when talking to a person of the opposite sex), SA10 (I rarely feel anxious in social situations), and SA15 (I feel relaxed even in a group of people who are quite different from me) were reverse scored.

### 4.2. Online social support

study used the online social support questionnaire developed by Liang and Wei (2008). The questionnaire includes four aspects: companion support, information support, emotional support and instrumental support, and the relevant contents were adjusted in the context of the research subjects in this study. Emotional support refers to the emotional communication and the care, encouragement and trust that can be obtained in online social interactions; instrumental support refers to the services or material help that can be obtained in online social interactions; information support refers to the information, advice or guidance that can be obtained in online social interactions; and buddy support refers to the sense of belonging to a group that individuals feel when they engage in online social or recreational activities. The scale consists of 23 questions, and is rated on a scale from “not at all consistent” to “completely consistent.” The higher the score, the more social support secondary school students have in the Internet.

#### 4.2.1. Working intensity

Work intensity was measured by including the number of hours worked and the amount of work tasks (Treuren and Fein, 2022), with a total of four topics to measure: hours worked per day (1 = less than 3 h, 2 = 3–5 h, 3 = 5–7 h, 4 = 7–9 h, 5 = more than 9 h); days worked per week (1 = less than 4 days, 2 = 4 days, 3 = 5 days, 4 = 6 days, 5 = 7 days); number of meetings per week (1 = less than 3, 2 = 3–5, 3 = 5–7, 4 = 7–9, 5 = greater than 9); and many work tasks per day (1 = completely disagree, 2 = disagree, 3 = average, 4 = agree, 5 = completely agree). Higher scores indicate more intense work (Table 1).

**Table 1:** Basic information of respondents.

Variable	Frequency	Percentage %
Gender
Male	125	32.6
Female	259	67.4
Age (years old)
From 20 to 30	48	12.5
From 31 to 40	216	56.3
From 41 to 50	47	12.2
From 51 to 60	73	19
Monthly income (RMB)
Below 3,000	8	2.1
3,000–5,000	121	31.5
5,000–7,000	174	45.3
7,000–1,0000	49	12.8
Above 10,000	32	8.3
Office mode
Online office	222	57.8
Offline office	162	42.2

### 4.3. Statistical analyses

Cronbach’s alpha coefficient was used to check the reliability of the data, and confirmatory factor analysis was used to check the validity of the data. Anova was used to analyze differences in social anxiety scores between demographic variables and work styles. Structural equation modeling is used to measure the proposed theoretical model and test the research hypothesis. Structural equation modeling has been widely used to test the relationship between multiple independent and dependent variables. It includes measurement model and structural model. Measurement models examine the relationship between latent variables and observed measurements, while structural models explain the structural relationship between latent variables. The purpose of multigroup analysis is to analyze whether the path model graph that can be applied to one group can also be applied to the corresponding model parameters of other samples. This study used a multi-group analysis to explore differences in patterns between online office and offline office. Multi-group analysis of structural equation modeling can evaluate whether the same theoretical model is consistently valid across groups or whether the parameters are constant. In this study, SPSS 24.0 and AMOS 24.0 were used for data processing, analysis and model fitting. In this study, *p* < 0.1 was considered significant.

The reason why SPSS 24.0 and AMOS 24.0 are used in this paper to process, analyze and model fit the data obtained from the questionnaire survey is that SPSS integrates data file management, statistical data editing, processing and analysis. Statistical analysis report generation, generation of various types of statistical tables, statistical programing and many other functions in one, covering all the commonly used statistical methods of statistics, especially the software is particularly suitable for the application of social economic statistics and management science of sampling survey data statistics and analysis, so this paper chooses this software to study the relevant issues for data statistical processing and analysis.

At the same time, AMOS is selected for the processing and analysis of survey data. Firstly, Amos can simultaneously process multiple simultaneous regression equations, that is, multidependent variable and multipath models, without the need of step processing like some other software, and output results in the form of the overall model graph with parameters. Secondly, Amos can test the overall fit degree of the model, such as GFI, RMSEA, etc., and can directly output the mediating effect and the total effect value. Thirdly, Amos can deal with latent variables, which is the outstanding advantage of the structural equation model compared with other models. Fourthly, Amos can conduct special problems such as multi-group analysis and constraint model testing. Fifthly, Amos is more convenient to construct model diagrams than other structural equation models. It is the most convenient software to construct and adjust model diagrams among current structural equation model software.

## 5. Results

### 5.1. Reliability and validity tests

As for the reliability of the scale, there are usually various methods to test the internal consistency of the scale, including Cronbach-α, covariance matrix, covariance matrix of multi-item scale, α and covariance matrix. Cronbach-α is the most commonly used test method for scale reliability. Cronbach-α coefficient ranges from 0 to 1, and the closer it is to 1, the better the reliability is. Its criterion is: coefficient above 0.9, indicating that the reliability of the measuring tool, namely the scale, is good. 0.8–0.9, indicating good reliability; 0.7–0.8 is acceptable, but some contents of the scale need to be modified; Below 0.7, it indicates that some contents of the scale need to be rewritten.

About the validity test of the scale, AVE, CR: The aggregate validity and discriminative validity of the indicators are mainly checked, and the output indicators include AVE and CR values. Generally, AVE value >0.5 and CR value >0.7 indicate that the data aggregation validity is good.

In this paper, SPSS 24.0 was used to analyze the research data, and the reliability of the data was tested by calculating the Cronbach’s Alpha value of the scale. The study showed that when the Cronbach’s Alpha value was greater than 0.7, the reliability of the data was good and met the analysis requirements. The results showed that the Cronbach’s Alpha of the Social Anxiety Scale was 0.938, the Cronbach’s Alpha of the Online Social Support Scale was 0.908–0.944, and the Cronbach’s Alpha of the Work Intensity Scale was The Cronbach’s Alpha of each scale was greater than 0.9, indicating that the reliability of the scale met the requirements.

In addition, the KMO values of 0.938 for the Social Anxiety Scale, 0.917 for the Online Social Support Scale, and 0.790 for the Work Intensity Scale were greater than the critical value of 0.7, indicating that the scale data were suitable for factor analysis. The structural validity of each scale was further examined using validated factor analysis, and the individual factor loadings were 0.541–0.964 for the social anxiety scale, 0.624–0.946 for the online social support scale, and 0.620–0.962 for the work intensity scale, with each factor loading greater than 0.5. In addition, this study also conducted a convergent validity test, based on the two main indicators of average variance extracted (AVE) and combined reliability (CR), and when AVE is greater than 0.5 and CR is greater than 0.7, it indicates that the scale has good convergent validity.

In conclusion, the reliability validity of the questionnaire data passed the test and can be further analyzed (Table 2).

**Table 2:** Reliability and validity tests results.

	Items	Cronbach’s α	KMO	Factor loadings	CR	AVE
Social Anxiety	15	0.964	0.938	0.541–0.964	0.943	0.537
Companion Support	7	0.930	0.917	0.624–0.946	0.928	0.654
Information Support	6	0.915			0.912	0.637
Emotional Support	6	0.944			0.929	0.691
Instrumental Support	4	0.908			0.912	0.723
Working Intensity	4	0.913	0.790	0.620–0.962	0.925	0.761

### 5.2. Variable description

**Figure 1:**
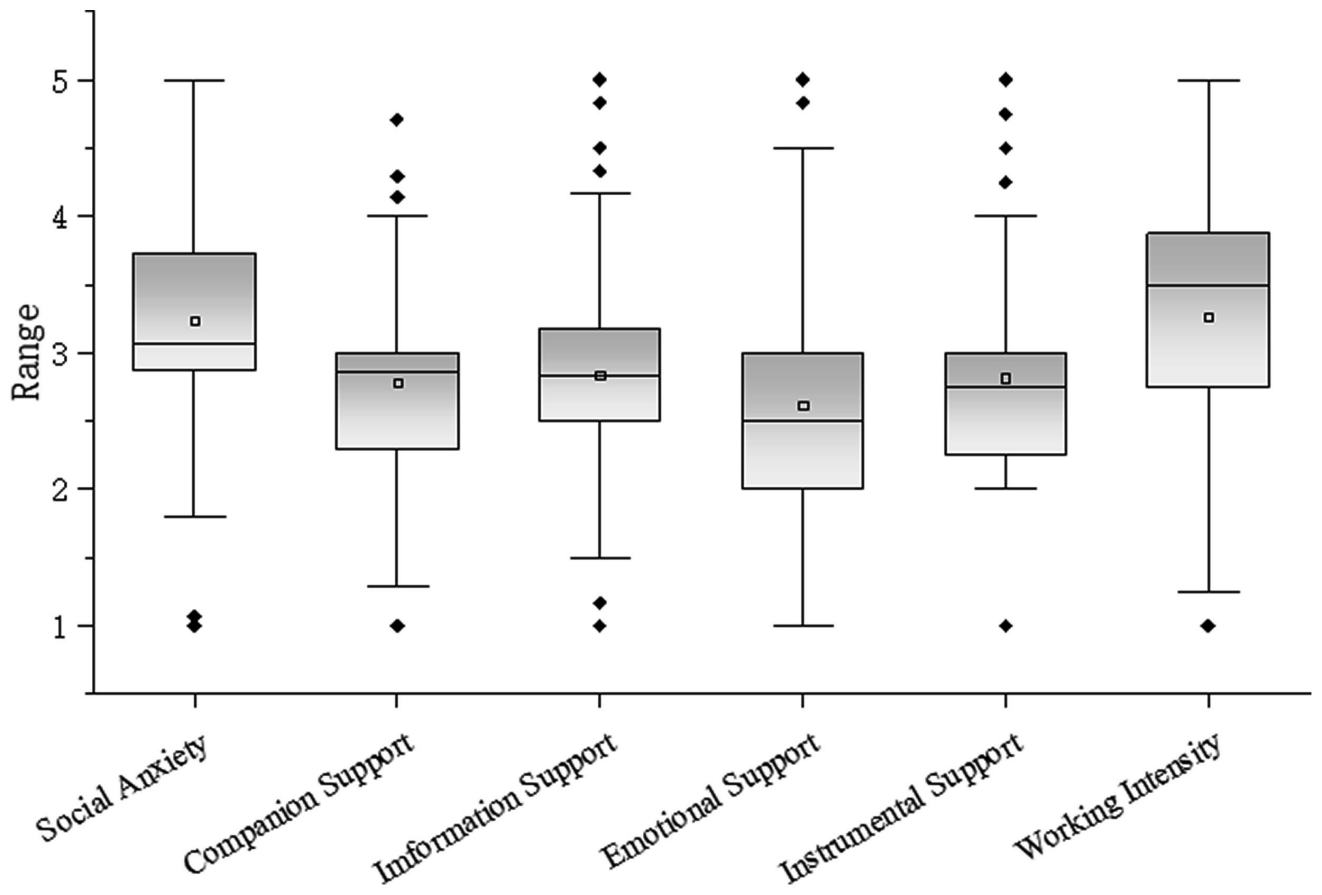
Score distribution of each variable.

As shown in Figure 1, the average of social anxiety is high, the scores are widely distributed, and there are a few abnormal values, indicating that the respondents’ social anxiety level is high and varies from person to person. The average value of companion support, information support, emotional support, and instrumental support is not much different, and they are all in the middle level. The normal value of emotional support is the most widely distributed, and the normal value of information support is the smallest. The average of work intensity is similar to social anxiety, and the median is the highest among all variables, indicating that half of the respondents have high work intensity.

### 5.3. Variance analysis

**Figure 2:**
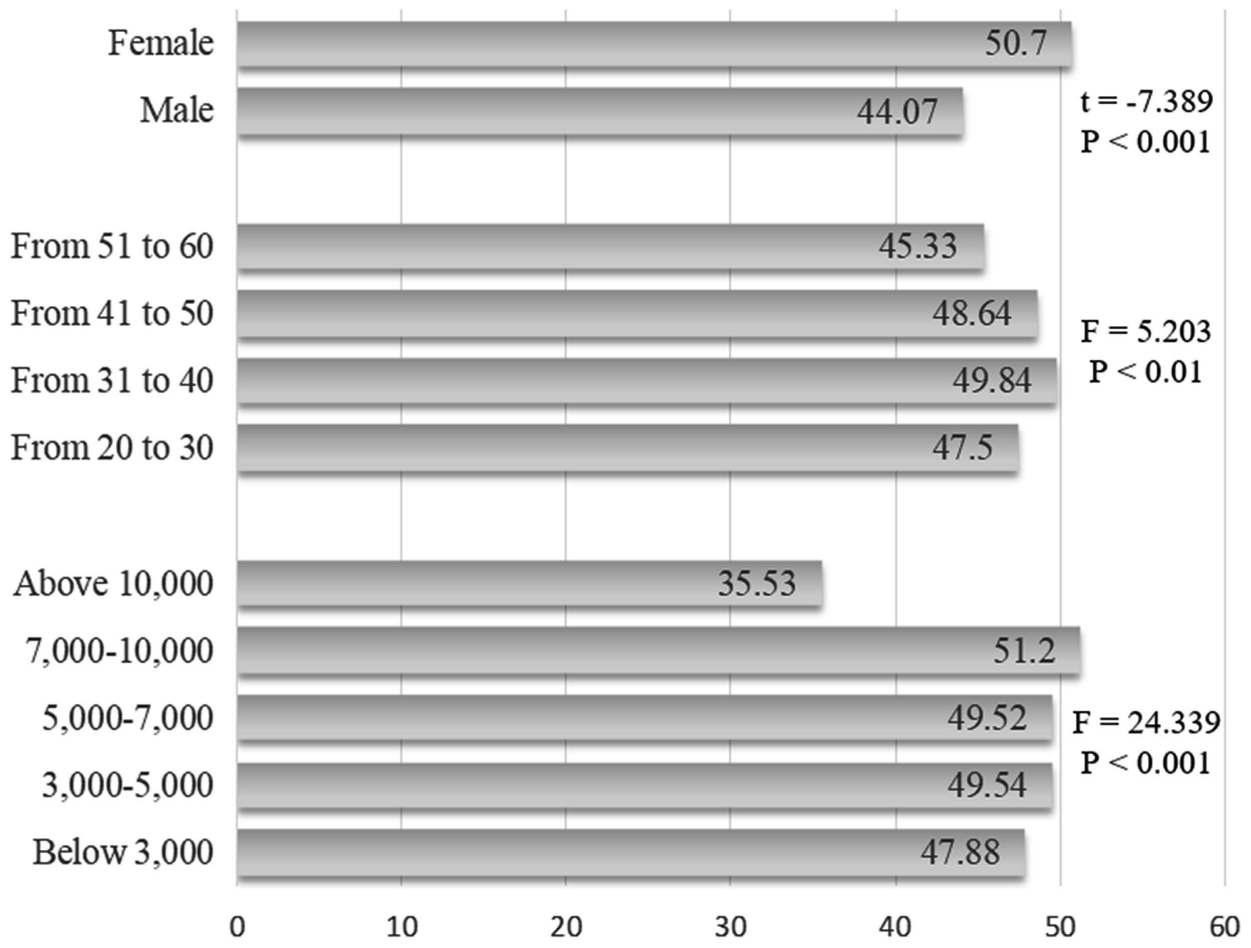
Differences in social anxiety across demographic variables.

The differences in social anxiety scores between genders, ages, and monthly incomes were compared by independent samples t-test and one-way ANOVA. As shown in Figure 2, social anxiety scores of primary and secondary school teachers differed significantly between genders, with females having significantly higher social anxiety than males’. Social anxiety scores differed significantly by age, with teachers between the ages of 31 and 40 having the highest social anxiety scores and teachers between the ages of 51 and 60 having the lowest social anxiety scores. Social anxiety scores differed significantly by monthly income, with teachers earning less than $3,000 per month having the highest social anxiety scores and teachers earning more than 10,000 per month having the lowest social anxiety scores.

Differences in social anxiety between work styles were compared by independent samples t-test. Overall, the total social anxiety scores of online office teachers were significantly higher than the social anxiety scores of office-based teachers. Specifically, online office teachers scored significantly higher on SA5 (parties often make me feel anxious and uncomfortable), SA6 (I may be shyer in social interactions than most people), SA7 (I often feel nervous when talking to people of the same sex whom I do not know well), SA8 (I get nervous in meetings), SA9 (I wish I had more confidence in social situations), SA13 (I usually feel more confident when calling people I do not know well), and SA14 (I often feel more nervous when calling people I do not know well), and SA15 (I feel relaxed even when I am in a group of people who are quite different from me) scored significantly higher than those of faculty in office settings (Table 3).

**Table 3:** Difference in social anxiety between telework and offline office.

Items	Telework	Office-work	*t*	*p*
SA1	3.38 ± 0.66	3.34 ± 0.79	0.582	0.561
SA2	3.41 ± 0.65	3.30 ± 0.84	1.345	0.180
SA3	3.39 ± 0.67	3.33 ± 0.84	0.832	0.406
SA4	3.41 ± 0.67	3.28 ± 0.82	1.584	0.114
**SA5**	**3.19 ± 0.72**	**3.00 ± 0.69**	**2.626**	**0.009**
**SA6**	**3.14 ± 0.74**	**2.89 ± 0.74**	**3.199**	**0.001**
**SA7**	**3.30 ± 0.68**	**3.13 ± 0.65**	**2.414**	**0.016**
**SA8**	**3.13 ± 0.74**	**2.88 ± 0.73**	**3.326**	**0.001**
SA9	3.40 ± 0.63	3.27 ± 0.73	1.864	0.063
SA10	3.41 ± 0.62	3.31 ± 0.73	1.395	0.164
SA11	3.33 ± 0.69	3.21 ± 0.78	1.634	0.103
SA12	3.39 ± 0.66	3.28 ± 0.72	1.444	0.150
**SA13**	**3.24 ± 0.69**	**3.01 ± 0.76**	**3.026**	**0.003**
**SA14**	**3.22 ± 0.67**	**3.01 ± 0.77**	**2.743**	**0.006**
**SA15**	**3.18 ± 0.67**	**2.99 ± 0.73**	**2.514**	**0.012**
**Total-SA**	**49.50 ± 8.12**	**47.23 ± 9.52**	**2.505**	**0.013**

In other words, respondents showed higher social anxiety in the teleoffice compared to the office-work.

### 5.4. Correlation analysis

**Figure 3:**
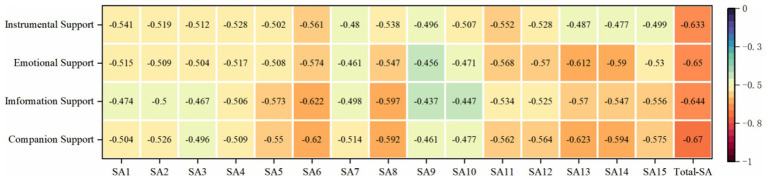
Results of correlation analysis between online social support and social anxiety.

Correlation analysis was used to analyze the degree of correlation between online social support scores and social anxiety scores, and the results are shown in Figure 3. The variables of online social support are significantly negatively correlated with social anxiety, and the variables of online social support have the highest correlation with the total score of social anxiety. Emotional support, information support and SA9 (I hope I can have more confidence in social occasions) have a low degree of correlation. The correlation between information support and SA10 (I seldom feel anxious in social occasions) is low. Companion support is highly correlated with SA6 (compared with most people, I may be shy in social interaction), SA8 (I am nervous at meetings), SA13 (I usually feel nervous when I call someone who is not familiar with me), and SA14 (I feel nervous when I talk with authority). The above analysis results provide a reference for further analysis of structural equation models.

### 5.5. Hypothesis testing

After the reliability and validity of the samples were affirmatively replied, the data were estimated by using the great likelihood estimation method of AMOS24.0 software on this basis to estimate the parameters, and then the degree of model fit was analyzed. After correction, the fit of the model met the requirements, and all the goodness-of-fit indicators showed that the model fitted the data well (as in Table 4). The chi-squared ratio of degrees of freedom (CMIN/DF) used to test the model fit was 3.245 < 5. The RMSEA, called the root mean squared of asymptotic residuals, is often considered the most important information about the model fit metric. Conventionally, if the RMSEA value is higher than 0.10, it is considered as poor model fitness; if the value is between 0.08 and 0.10, it indicates fair model fitness; if the value is between 0.05 and 0.08, it indicates good model fitness; if the value is less than 0.05, it indicates very good model fitness. The RMSEA value in this study is 0.039, which indicates that the model has good fitness. The GFI and PGFI are greater than 0.6, and the NFI, IFI, TL, and CFI all meet the best fitness criterion of 0.9.

**Table 4:** Model fitting indicators.

CMIN/DF	RMR	GFI	PGFI	NFI	IFI	TLI	CFI	RMSEA
3.245	0.049	0.810	0.633	0.912	0.908	0.921	0.914	0.039

In this study, it is concluded that the research hypothesis of the path in the model is valid when the significance level of the coefficients of a path are accompanied by probability *p*-values below 0.1, and it is concluded that the research hypothesis of the path in the model is not valid when the significance level of the coefficients of a path are accompanied by probability *p*-values greater than 0.1.

**Figure 4:**
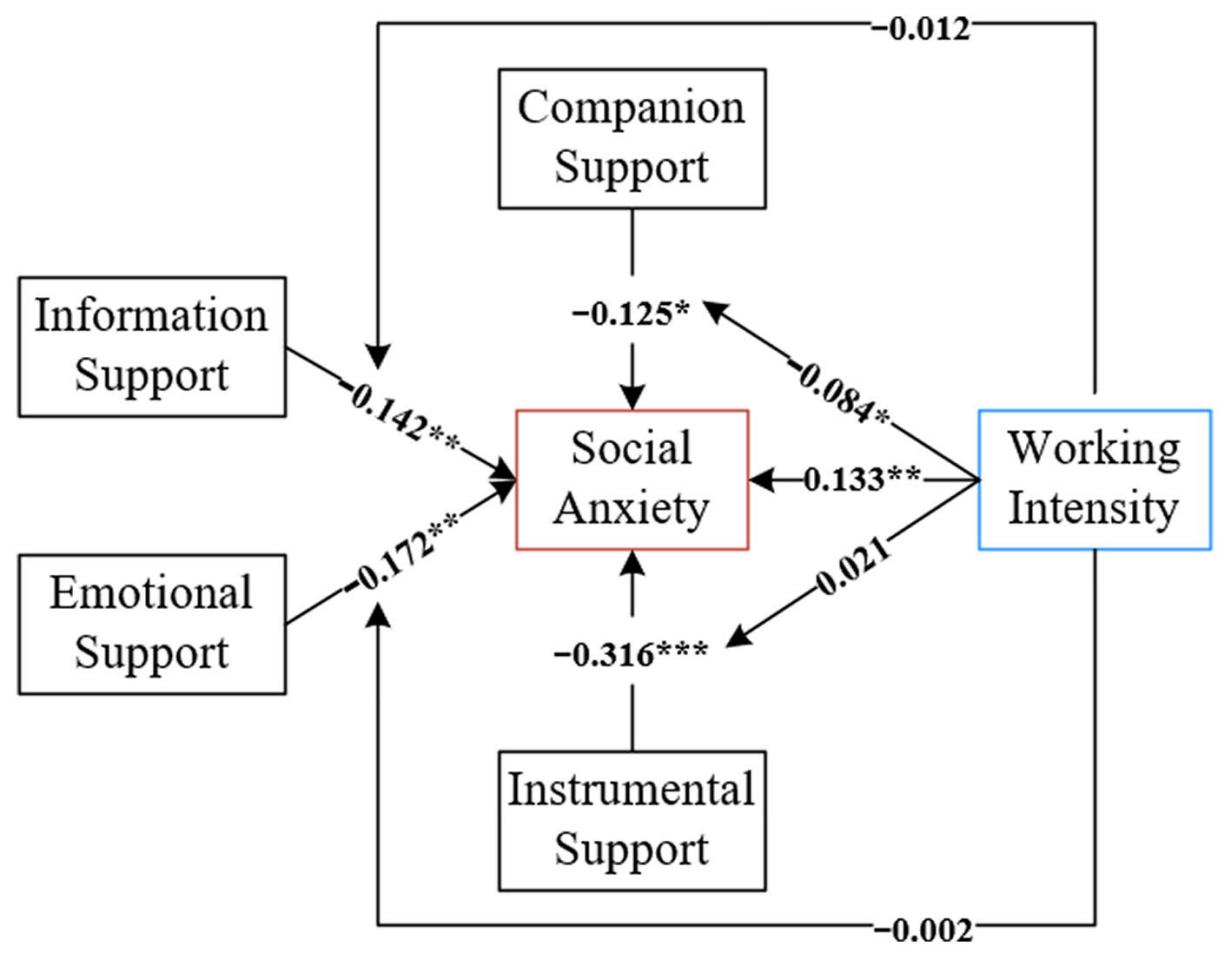
Results of the structural model. *p < 0.01; **p < 0.05; ***p < 0.01.

The results are shown in Figure 4. Without distinguishing between online office and offline office, there was a significant positive effect of work intensity on social anxiety, indicating that the higher the work intensity, the more severe the teachers’ social anxiety condition, and hypothesis H1 holds. The hypothesis H2 holds that there is a significant negative effect of companion support on social anxiety; the hypothesis H3 holds that there is a significant negative effect of information support on social anxiety; the hypothesis H4 holds that there is a significant negative effect of emotional support on social anxiety, and the teachers’ anxiety condition will be improved; the hypothesis H5 holds that there is a significant negative effect of instrumental support on social anxiety; it indicates that the more online social support, the teachers’ social The more online social support, the more teachers’ social anxiety will be improved. Work intensity only has a negative regulatory effect between companionship support and social anxiety, assuming that H6 is true, H7, H8, and H9 are not.

### 5.6. Multi-group analysis

**Figure 5:**
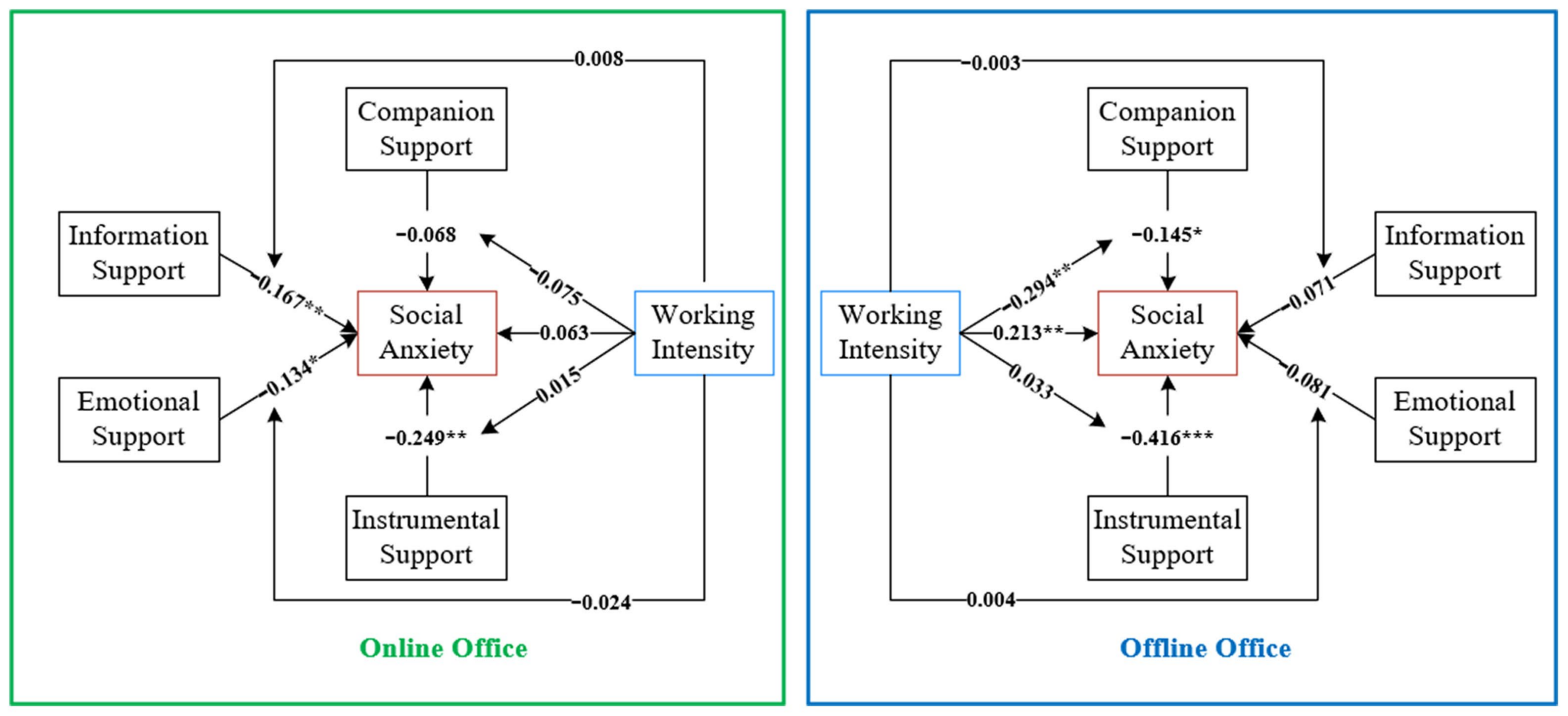
Differences of models under different office modes. *p < 0.01; **p < 0.05; ***p < 0.01.

A multi-cluster analysis on AMOS was used to explore the variability of the model between online office and offline office and to provide insight into the mechanism of the effect of online office modalities on social anxiety among elementary and middle school teachers. The results are shown in Figure 5. There were significant differences in the model between online office and offline office, as evidenced by:

The positive effect of work intensity on social anxiety was not significant for online office (*β* = 0.063, *p* > 0.1) and significant for offline office (*β* = 0.213, *p* < 0.05).

The negative effect of companion support on social anxiety was not significant on online office (*β* = −0.068, *p* > 0.1) and significant on offline office (*β* = −0.145, *p* < 0.1).

The negative effect of information support on social anxiety was significant for online office (*β* = −0.167, *p* < 0.05) but not for offline office (*β* = −0.071, *p* > 0.1).

The negative effect of emotional support on social anxiety was significant for online office (*β* = −0.134, *p* < 0.1), but not for offline office (*β* = −0.081, *p* > 0.1).

The negative effect of instrumental support on social anxiety was significant for online office (*β* = −0.249, *p* < 0.05) and for offline office (*β* = −0.416, *p* < 0.01).

Under online office mode, there is no moderating effect between the four variables of online social support and social anxiety; In offline office mode, work intensity only has a negative regulatory effect between companion support and social anxiety.

## 6. Discussion

The aim of this study was to investigate the mechanisms of the effect of teleworking style on social anxiety of elementary and secondary school teachers, by working with non-teleworking style and considering online social support and work intensity. The results of the study showed that primary and secondary school teachers with teleworking styles had significantly higher scores of social anxiety than those with non-teleworking styles, and similarly, showed similar results in other groups (Barros, 2017). Teachers, as a special group, have the burden of “teaching and educating” in terms of their social role and have more anxiety than the general population in their ordinary lives (Wang et al., 2010). During an epidemic, teachers are often exposed to pictures, videos, and texts about the epidemic, which inevitably lead to negative emotions such as sadness, anxiety, and fear, as well as anxiety about the need for safety in life and the unpredictability of their current and future lives. As the epidemic progresses, both the community and the school are in a state of shutdown, and the prolonged home office makes teachers all feel anxious and overwhelmed.

Female teachers had significantly higher social anxiety scores than male teachers, consistent with the findings of studies on factors influencing teacher anxiety (Pressley et al., 2021).In other words, female teachers are more likely to experience social anxiety, and more attention should be given to female teachers when conducting psychotherapy. Teachers aged 30–40 are more likely to experience social anxiety because teachers in this age group take on more work and are at a critical time in their careers when work takes up most of their leisure time and social time. In addition, teachers with lower incomes are more likely to experience social anxiety, and economics is an important factor that affects people’s physical and mental health (Satoshi Kuhara et al., 2022). Therefore, giving more financial support to primary and secondary school teachers is beneficial in reducing social anxiety and thus promoting the quality of educational work.

As shown in Table 2, work intensity has a significant positive effect on social anxiety, meaning that the higher the intensity of teachers’ work, the more likely it is to lead to social anxiety, which is consistent with the findings of Satoshi Kuhara et al. (2022); Semple (2021) and Treuren and Fein (2022). Higher work intensity takes away from physical activity and social time, which negatively affects teachers’ physical and mental health and is detrimental to labor sustainability and disease risk management. Despite some government policies on reducing the burden of primary and secondary school teachers (Satoshi Kuhara et al., 2022), the work intensity of primary and secondary school teachers remains high due to the specificity of the profession and the continuous competition in the education industry. Moreover, teachers’ work has long been characterized by blurred boundaries, and weak work boundaries have led to a lack of clarity in the division of teachers’ “work time” and “rest time” (Liu and Yang, 2022). In addition, teachers’ invisible workloads are numerous, and many non-teaching “invisible tasks” consume a lot of teachers’ time and energy (Ma and Li, 2013; Semple, 2021). Therefore, rationalizing teachers’ workload and working hours can effectively reduce teachers’ social anxiety.

Online social support was effective in reducing the probability of social anxiety among primary and secondary school teachers, in line with the findings of Mohammed Feroz Ali et al. (2021); Huang et al. (2017). Specifically, companion support, emotional support, informational support, and instrumental support all had a significant negative effect on teachers’ social anxiety; in other words, the more online social support elementary and secondary school teachers received, the less likely they were to develop social anxiety. It has been shown that social support provided by close interpersonal relationships can help teachers relieve stress in many forms, including regulating adverse emotions (anxiety, depression, etc.), providing advice and help, etc. (Mohammed Feroz Ali et al., 2021). For teachers, family, relatives, and friends can not only provide love and care for his/her inner world, but also enable college students to gain emotional security and support to provide a basis for social competence, thus improving social anxiety and reducing social avoidance behaviors. Especially during an epidemic, working from home prevents teachers from engaging in offline communication activities, and online social support can improve just that. Therefore, enhancing online social support for primary and secondary school teachers, such as providing some useful information, helpful materials, or emotional care, can effectively improve teachers’ social anxiety.

A multi-cluster analysis revealed that the model’s path coefficients differed significantly between remote working and non-remote working modes, as shown by:

First, the effect of work intensity on social anxiety was not significant for teleworking, but was significant for non-teleworking. This may be due to the fact that teachers who use teleworking are isolated at home for long periods of time and are unable to engage in offline face-to-face communication regardless of work intensity, which leads to social anxiety. In contrast, the social activities of teachers who used non-teleworking methods were severely affected by the intensity of their work, and when their work tasks were light and they had sufficient time for leisure activities, they could engage in social activities to reduce social anxiety. In addition, work intensity can weaken the influence of companion support on social anxiety. In other words, the higher the work intensity, the lower the effect of companion support on social anxiety. The moderating effect of work intensity is different under different office modes. When working online, there is no moderating effect between companion support and social anxiety. This is because teachers who online office often stay with family and friends, and the effect of companion support is not affected by work intensity. On the contrary, when offline office takes up most of teachers’ time and energy, the effect of family and friends’ company on social anxiety will not be so obvious.

Second, the coefficient of the effect of buddy support on social anxiety was not significant for telework but significant for non-telework. Under normal circumstances, the more companionship a teacher receives, the lower the social anxiety will be. However, the effect of peer support is diminished when teachers who work remotely live with family or colleagues for long periods of time.

Third, the coefficient of the effect of information support on social anxiety was significant for remote work but not for non-remote work. This is due to the fact that teachers often deal with colleagues, leaders, and students when they work offline, so information transmission is more timely and frequent, and such regular information support does not cause psychological fluctuations in teachers and therefore does not have a significant effect on social anxiety. On the contrary, teachers in a teleworking state can only communicate with the outside world through electronic devices, and information transmission will be lagging or even occluded, such as ignoring a meeting notice or an important notice, which will cause psychological stress and anxiety to teachers.

Fourth, the coefficient of the effect of emotional support on social anxiety was significant for remote work but not for non-remote work, for reasons similar to the mechanism of action of information support. Online emotional support is reflected in the emotional help that teachers can access through the Internet, such as others’ responses to their statements, congratulations from others, recognition from others, and emotional empathy. When teachers work offline, others are able to give these emotional supports back to them in a timely manner.

Fifth, the effect of instrumental support on social anxiety was significant both on telework and non-telework, suggesting that instrumental support is an important factor influencing social anxiety. Instrumental support reflects the materials, items, etc. that teachers can access on the internet. In Chinese society, people in epidemic-controlled areas receive material support from other places and institutions, reflecting a spirit of solidarity and love, which is a great boost to people’s spirits and helps to improve the anxiety of people in home isolation. Similarly, the more instrumental support primary and secondary school teachers receive when working from home, the less likely they are to develop social anxiety. When teachers are not working remotely, materials and objects are not as timely as information and emotional support, so the importance of instrumental support for social anxiety is not affected by timeliness.

Sixth, previous studies on remote and non-remote working social anxiety of primary and secondary school educators and its influencing factors are based on the general background, while this study in this respect is aimed at the special background of the impact of the epidemic. Due to the particularity of this background, As a result, the influence of work intensity, partner support, emotional support and other factors on social anxiety of primary and secondary school educators has new characteristics, so that this paper has reached a new conclusion in this research.

Previous studies on social anxiety of primary and secondary school educators and its influencing factors usually separate remote working and non-remote working of primary and secondary school educators, and rarely make a comparative analysis of remote working and non-remote working. However, this paper makes a breakthrough in this aspect, and makes a comparative analysis of the two. The difference between the two can be more clearly understood.

Previous studies on the social anxiety of primary and secondary school educators in telecommuting or non-telecommuting and its influencing factors mostly studied the direct impact of independent variables on dependent variables, and few considered the introduction of moderating variables. However, in this study, relevant moderating variables were introduced to make the study closer to reality.

## 7. Conclusion

### 7.1. Practical theoretical

The aim of this study was to investigate the mechanisms underlying the effects of teleworking styles on social anxiety among primary and secondary school teachers, while considering the effects of online social support and work intensity on teachers’ social anxiety. Based on 384 valid questionnaire data, this study used structural equation modeling and multi-group analysis to provide insight into the factors influencing social anxiety among primary and secondary school teachers, as well as the differences in social anxiety between remote working styles compared with non-teleworking in the context of the COVID-2019 pandemic. The results found that teachers’ social anxiety was significantly higher in the teleworking mode than in the non-teleworking mode. There were significant differences in teachers’ social anxiety profiles between gender, age and monthly income. The intensity of the work facilitated teachers’ social anxiety, while the network support was effective in improving teachers’ social anxiety. Work intensity weakens the influence of companion support on social anxiety. Finally, the model’s path coefficients differed significantly across work styles. Overall, teachers who work remotely should be given more online social support, such as information support, emotional support and instrumental support, while work intensity should be rationalized and more online communication activities should be organized to improve teachers’ communication mood. The findings of this study have important implications for the sustainable development of educational labor and for improving the physical and mental health and quality of work of primary and secondary school teachers.

### 7.2. Limitation

1. Due to the constraints of the epidemic, the data supporting this study mainly came from the online survey format, which affected the authenticity and reliability of the data to a certain extent. In future studies, if the survey conditions permit, the offline method can be further considered to obtain more reliable offline survey data, so as to make the research results more convincing.
2. Limited by the research time and length, the analysis of the mechanism of remote work on the social anxiety of primary and secondary school teachers, as well as the mechanism of the influence of network social support and work intensity on the social anxiety of teachers is not in-depth enough. Further in-depth analysis of this aspect can be considered in the future.

### 7.3. Future studies

1. This paper focuses on the influence mechanism of remote working mode on social anxiety of primary and secondary school teachers, and also considers the influence of network social support and work intensity on teachers’ social anxiety. However, these studies mainly consider the direct influence of relevant independent variables on dependent variables. In future studies, some mediating variables can be further considered. Then how the independent variable influences the dependent variable through the intermediary variable makes the research closer to the reality of the problem.
2. Future studies can further consider the influence of other factors other than remote working mode, network social support and high wage intensity on the reduction of social anxiety in primary and secondary schools, so as to make the research issues more comprehensive.

## Data availability statement

The original contributions presented in the study are included in the article/supplementary material, further inquiries can be directed to the corresponding author.

## Author contributions

Yating Xie: methodology, data analysis, writing, writing-reviewing and editing, article review, and intellectual concept of the article.

## Conflict of interest

The author declares that the research was conducted in the absence of any commercial or financial relationships that could be construed as a potential conflict of interest.

## Publisher’s note

All claims expressed in this article are solely those of the authors and do not necessarily represent those of their affiliated organizations, or those of the publisher, the editors and the reviewers. Any product that may be evaluated in this article, or claim that may be made by its manufacturer, is not guaranteed or endorsed by the publisher.
